# Demographic characteristics shape patterns of dawn swarming during roost switching in tree-dwelling Daubenton’s bat

**DOI:** 10.1038/s41598-022-14246-2

**Published:** 2022-06-15

**Authors:** Romana Ružinská, Denisa Lőbbová, Peter Kaňuch

**Affiliations:** 1 Institute of Forest Ecology, Slovak Academy of Sciences, Zvolen, Slovakia; 2 Faculty of Ecology and Environmental Sciences, Technical University in Zvolen, Zvolen, Slovakia; 3 Slovak Bat Conservation Society, Bardejov, Slovakia

**Keywords:** Behavioural ecology, Animal behaviour

## Abstract

Frequent roost switching in fission–fusion societies of tree-dwelling bats is closely associated with swarming behaviour entailing ritualised night-time displays around the roost tree and/or at the roost entrance to signal its actual location, particularly immediately prior to sunrise. However, effects of demographic characteristics of individuals in this social behaviour remain unanswered. Using passive integrated transponders (PIT) and automatic readers, we recorded swarming activity of members of a Daubenton’s bat (*Myotis daubentonii*) maternity colony in the vicinity of their roosts. In total, 59,622 activity events of 281 PIT-tagged individuals were recorded on ten monitored roosts during three summer seasons. We found a gradual increase of swarming activity from midnight to sunrise in old adult females, whereas young females and juveniles primarily swarmed later at dawn. We attribute this difference to the learning status of younger bats, which are not yet able to perform a defined pattern of swarming activity, whereas older bats likely take a more active role in signalling the position of the roost. Old males exhibited the least swarming activity at maternity roosts, which mostly occurred between crepuscular periods, presumably due to their solitary lives. A negative correlation between genetic distance and swarming activity suggests an important role of kinship in the formation of the maternity colony as well as group cohesion during roost switching.

## Introduction

Animal decision-making requires the gathering and processing of information from their surroundings. Whether such information is abiotic (e.g. location of the potential shelter or some resources) or biotic (often presence of competitors or a predator), it includes a social environment represented by signals from conspecifics^1^. Several activities, movements or structures serve as signals, although some of them had originally different purposes and acquired signalling function only by a ritualization. For the successful process of signalling, a receiver has to notice a correlation between the signal and actions of a transmitter. For enhancing an intensity of a signal, ritualization takes an advantage of stereotypization and thus, increasing its conspicuousness and separate behaviour from its original function^2^. The most frequent example of ritualization in bats is aggressive territorial behaviour, as was reported in Seba’s short tailed fruit bat (*Carollia perspicillata*)^3^, however, ritualised behaviour was observed also to be a part of promiscuous mating system as a swarming activity at bats’ underground hibernacula^4^. Additionally, ritualization extended the function of a swarming behaviour even more.

In 1976, Vaughan and O’Shea^5^ described a ritualised swarming behaviour (hereafter ‘swarming’ or ‘dawn swarming’) in tree-dwelling Pallid bats (*Antrozous pallidus*) during dawn period before sunrise. Thus, this behaviour has long been anecdotally known^6^; however, its function has only been analysed in the last decades. Whereas increased levels of swarming activity positively enhance cohesion of social groups within colonies^7,8^, it was assumed serving as a means of information exchange about bats’ intention to switch roosts in non-centralised decision-making process^9^. This is especially important due to the fission–fusion dynamics of bat societies, whereby colony members freely move between multiple smaller social groups and are also able to fuse to a large unit^10^. Thus the main reason of frequent roost switching in tree-dwelling species in which a set of roosts is re-used is thought to be mainly social, i.e. to maintain social bonds among a larger number of animals that cannot fit in a limited space of tree roost as found for example in big brown bats (*Eptesicus fuscus*)^11^. Maternity colonies of these bats tend to switch roosts every 2–5 days or even daily^12^; therefore, efficient information transfer among individuals is needed to prevent group disintegration^8,9^.

Dawn swarming is based on various behavioural components, including flybys, landings, leaps, crawling, entrances and departures. In noctule bats (*Nyctalus noctula*, *N. leisleri*), this behaviour was recently found to be performed throughout most of the night but mostly prior to sunrise around the roosting tree and/or at the roost entrance^7,8^. Cooperating individuals are expected to act as both signallers and receivers that communicate the locations of potential roosts within the area of a colony home-range. Besides maintaining social bonds, switching of roosts is likely triggered also by unsuitable conditions in the current tree roost due to unsuitable microclimatic conditions^13^ and/or parasite overgrowth^14^; however, it may serve as a strategy to avoid predators^15^. Although individuals may intersperse with bats from other social groups^16^, relationships between females of tree-dwelling species within the defined social group remain preserved. Studies examining the social structures of maternity colonies have shown that some female pairs with a higher degree of kinship may roost together more frequently over a long time^17^. More related individuals thus express similar personality traits in their behaviour^18,19^. Therefore, individual genetic distance to the rest of the colony may also affect cooperative behaviour associated with roost switching and social grouping.

However, there are demographic characteristics of individuals which need to be considered first when analysing dawn swarming behaviour. The most prominent are sex- and age-related differences in social behaviour of bats. Sex-based behavioural differences have been found to be mostly shaped by natural selection and constrained by factors such as environment, endocrine status or reproductive condition^20,21^. Males and females of socially organised mammalian species have normally different social roles, which correspond with varying behaviours^22^. Among bats living in temperate zones, females typically segregate from males in spring after hibernation to form breeding colonies with stable social structures in which an individual strongly depends on their conspecifics^23^. Whereas most temperate zone male bats live solitarily or in bachelor groups during maternity colony formation^24^, the occurrence of males in the maternity colony home range could be explained by a shortage of roosting options or mating opportunities there^25,26^. These lifestyle differences suggest sex-related characteristics of swarming behaviour^20^. Many mammals display sex-related behavioural variations from a young age, and these differences are linked with adult fitness-correlates^27^. However, age-related changes in animal behaviour have also been observed. Some aspects of juvenile behaviour are specifically adapted for their survival and propagated during specific period only (e.g. food begging), whereas others are precursors of adult behaviour as a play is. Typically, the development of juvenile behaviour depends on an interplay between inherent elements and learning^28^. Social learning among bats has been demonstrated in food related cues^29^, vocalisation^30^, and roost localisation^31^.

Demographic characteristics of individuals and their mean relatedness to maternity colony may shape also cooperative behaviour which maintains cohesion in social groups of bats. This study aimed to understand the role of sex, age and individual genetic distance from other colony members in dawn swarming of tree-dwelling Daubenton’s bats during roost switching. We examined three hypotheses: (1) Adult females that remain with their relatives throughout their lives will evince increased dawn (nocturnal) swarming activity to enhance social bonds in maternity colonies. Contrary to this, adult males which do not depend on the energetic benefits of maternity groups during nursing will be less active in swarming. (2) Swarming activity patterns will change throughout the lifespan, especially in adult females as a result of social learning. (3) Individual genetic distance will have an impact on the level of swarming activity as the kinship may support roosting together. To study individual variation in swarming activity according to sex, age and genetic distance, we tracked members of a maternity colony (adult females and juveniles with an associated proportion of adult males) of Daubenton’s bats using passive integrated transponders (PIT tags) and a set of automatic PIT tag readers that recorded bats’ activity at tree roost entrances.

## Materials and methods

### Study species

The Daubenton’s bat (*Myotis daubentonii*) is a medium-sized bat weighing 6–10 g with a forearm length of about 40 mm that is common in most of Europe^8^. This species specialises on chironomids, which are foraged over calm water surfaces by trawling or aerial hawking^32,33^; thus, its summer occurrence is strictly bound with riparian stands, where it mainly roosts in various tree cavities, although maternity colonies also rarely occupy rock shelters or man-made structures^34,35^. The species has a promiscuous mating system, and a relatively high proportion of adult males has been recorded in maternity colonies^25,26^. The Daubenton’s bat is considered a short-distance migrant with stable social groups^12,36^ and a relatively long lifespan of about 4.5 years on average^37^.

### Study area

**Figure 1:**
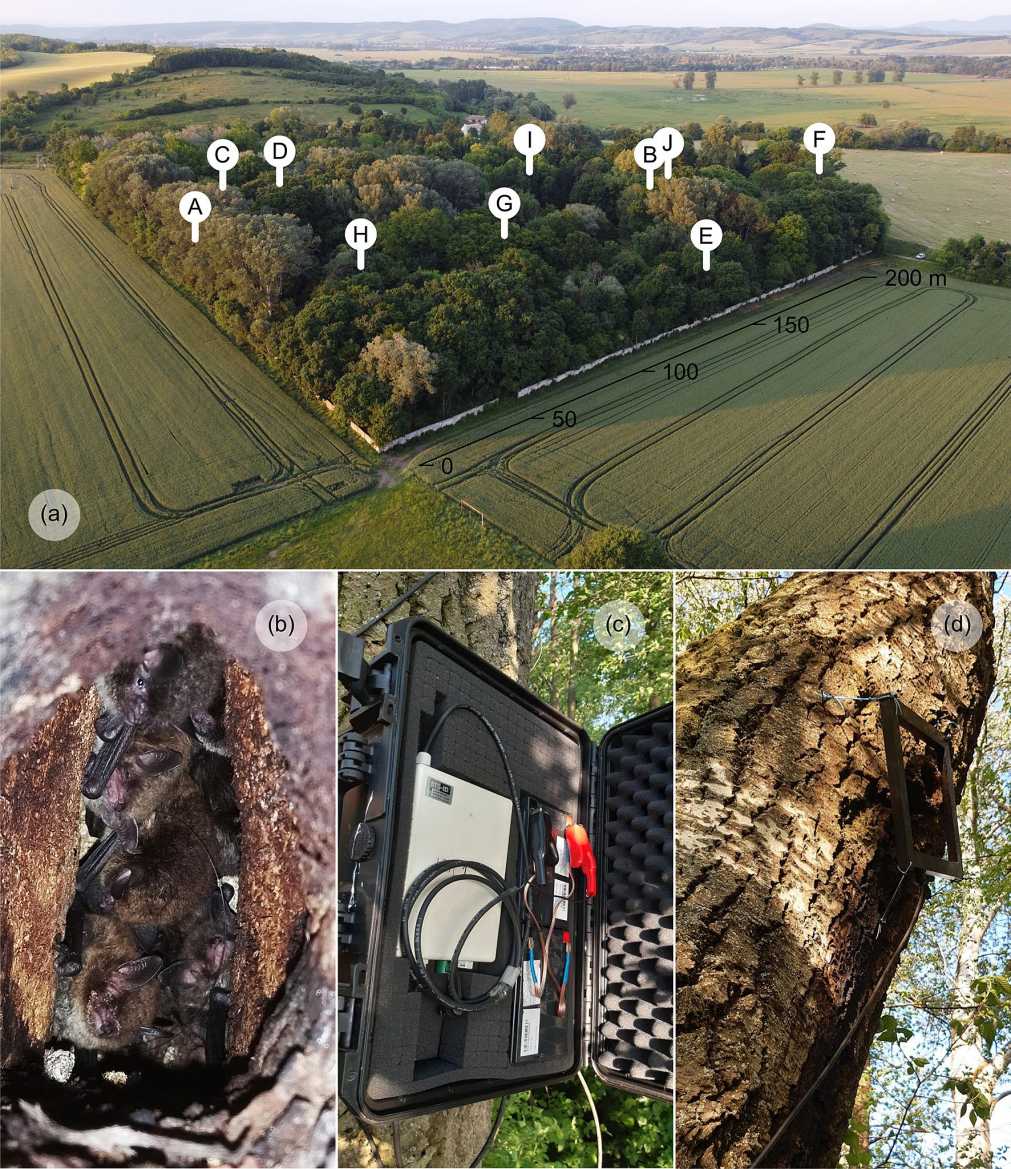
(**a**) The study area with locations of tree roosts of the Daubenton’s bat in an old park in southern Slovakia. (**b**) Maternity colony of Daubenton’s bats in a tree roost. (**c**) Automatic PIT tag reader protected by waterproof case with (**d**) square-shaped antenna placed over roost entrance (Photo by P. Kaňuch).

The study was conducted at Čeláre–Kirť, a small park (~ 7 ha) located in southern Slovakia (Ipeľská Kotlina Basin): 48.116464 N, 19.489140E, 151 m a.s.l. (Fig. 1a). The park is surrounded by an agricultural landscape, and most of it comprises the remnants of an old hardwood floodplain forest. Dominant tree species are native willows (*Salix alba*), poplars (*Populus alba*), maples (*Acer platanoides*), oaks (*Quercus robur*) and ashes (*Fraxinus excelsior*), and there are also apple trees (*Malus* sp.), horse chestnuts (*Aesculus hippocastanum*) and sycamores (*Platanus* sp.). A dense undergrowth is formed by self-seeding vegetation and various deciduous shrubs and bushes. The Ipeľ River and riparian stands are located east- and southward from the park. The park is connected with the river by a corridor of tree vegetation, which bats use for navigation to the foraging area during their evening emergence. In addition to Daubenton’s bats (Fig. 1b), Natterer’s bats (*Myotis nattereri*) and Common noctules (*Nyctalus noctula*) have been documented breeding in the park’s trees^34^, and Alcathoe bats (*Myotis alcathoe*), Grey long-eared bats (*Plecotus austriacus*), Soprano pipistrelles (*Pipistrellus pygmaeus*) and Serotines (*Eptesicus serotinus*) have also been recorded there.

### Recording of bat swarming activity

**Figure 2:**
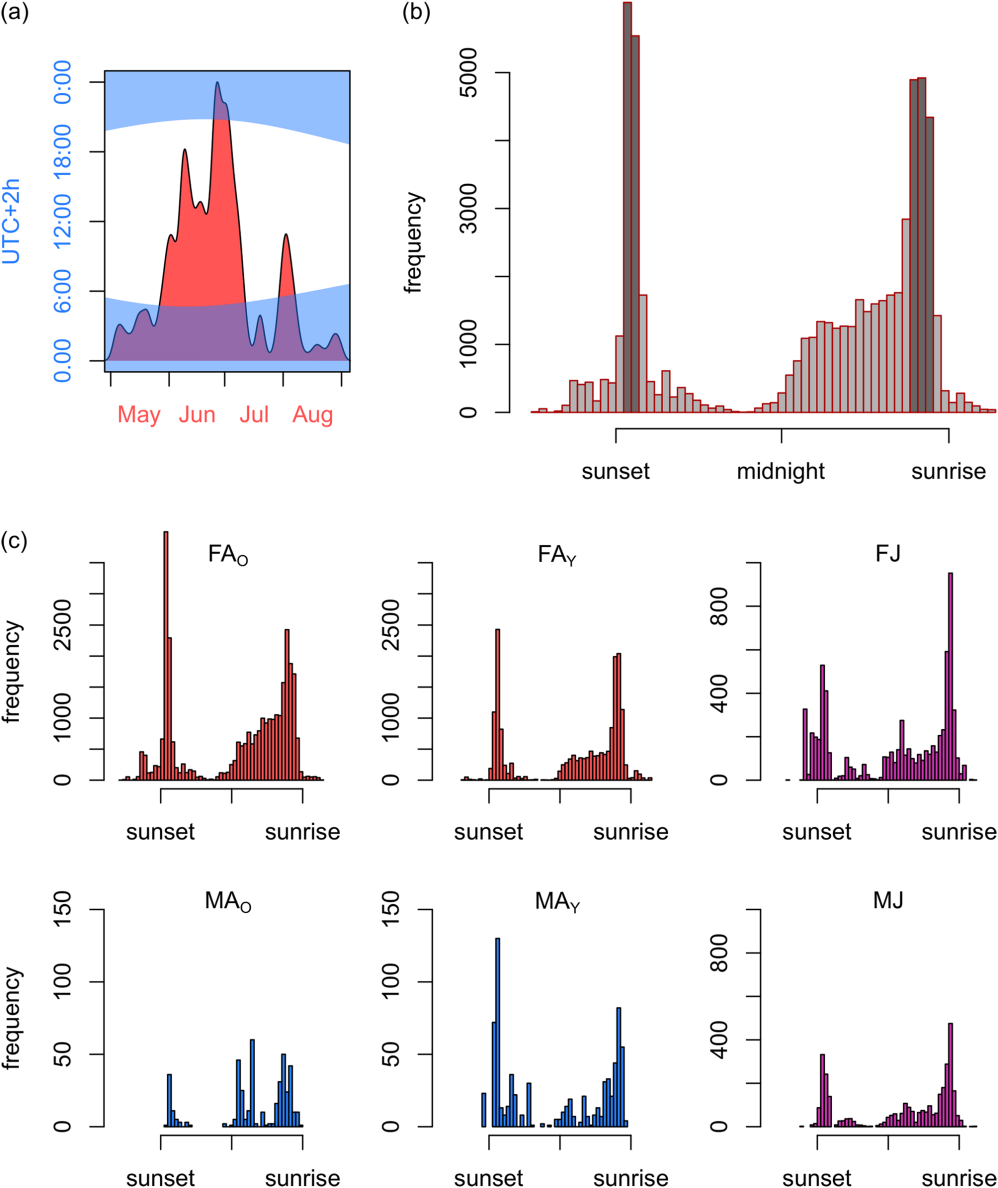
(**a**) Density distribution of records of bat behavioural events during summers 2019–2021 with the outlined length of the darkness during this study period. Frequency of behavioural events from all seasons of (**b**) all individuals and (**c**) individuals categorised by sex and age relative to the sunset and sunrise, respectively (codes: F = female, M = male, A = adult, O = old, Y = young, J = juvenile; categories are also colour coded). Events include any presence of bats at the cavity entrance, mainly emergence, swarming and entering the tree. Each bar in the histograms represents approximately 15 min (note: night length during season differed but most events were recorded around midsummer with minimal variation in the night length).

During two breeding seasons (from May to August in 2019 and 2020), we equipped a set of 12 mist-netted bats (preferentially older adult females) with transmitters (PicoPip Ag317, weight 0.4 g; Biotrack/Lotek, Inc.) and located their roosts using TRX100 S3 receiver and a three-element hand-held directional Yagi antenna (Wildlife Materials, Inc.). Some roosts were opportunistically found during morning observations of swarming behaviour in the park; however, most were identified through radio-tracking. Simultaneously, we made mist-netting sessions approximately twice a week on the commuting corridor, and all animals were labelled with individual identification codes by subcutaneous insertion of PIT tags (ID100 Mini Transponder, diameter 1.4 mm, length 8 mm; Trovan, Ltd.) in their scapular region^38^. In total, 389 bats were PIT-tagged in the area. Along with PIT-tagging we installed automatic PIT tag readers (BTS-ID, Sweden; Fig. 1c) on altogether 10 bats roosts in tree hollows during three consecutive seasons from May 2019 to early September 2021. The number of monitored roosts differed between seasons according to our knowledge about their occupancy by adult females and their offspring (4 roosts were successfully monitored in 2019, 8 in 2020 and 7 in 2021). The PIT tag readers antennas placed over roost entrances were square-shaped and measured 30 × 30 cm (Fig. 1d). Due to technological limitations, this approach may not provide accurate determination of all displays of swarming behaviour, which is mainly performed around the tree outside the distance recordable with antennas; however, it can provide reliable summary measures of most events (landings, leaps, crawling, entering and departures). We used such events as a proxy of relative bat activity at the roost. The device’s reading sensitivity allowed a setting of 1 s as the minimal time for one event, thus all bats which crossed a PIT tag reader antenna frame or occurred at its close vicinity were recorded (transponders were recognisable outside the frame of antenna at a distance of 2 cm when tested). Some multiple events of a same individual repeatedly recorded in continual sequence every second for up to few minutes (e.g. bat did not swarm, but rather sat in the entrance hole) were deleted from data, presumably indicating behaviour without information value for the rest of the colony or some technical error. This represented less than 1% of all data. However, simple record from each such multiple session was maintained. Although records of evening departures from and final entrances to roosts are not displays in the sense of swarming, they were left in the dataset under the assumption that these result in minimal bias in swarming patterns. In our setup of PIT tag readers fixed on limited number of roosts, the amount of recorded activity that we finally collected differs throughout the season. Most activity was recorded in June and early July during the nursing of juveniles before they were weaned from the maternity colony (Fig. 2a). First flights of juveniles started in early July. During nursing period when females return also around midnight to feed juveniles, thus make an extra entrance and departure^39^, we could expect some effect in seasonal distribution of data. However, we accepted this possible bias as insignificant, considering the fact that analysed data presented events pooled from all three years. Therefore we were confident that our robust data set from all three seasons was suitable to analyse the role of demographic characteristics in swarming behaviour of bats during roost switching.

In addition to sex, the age of all PIT-tagged individuals was determined on the basis of phalangeal epiphysis^40^ and chin-spot colouration^41^. Individuals with entirely cartilaginous epiphysis were classified as juveniles, whereas bats with completely ossified joints were sorted between ‘young adults’ (dark or intermediate chin spot) and ‘old adults’ (no spot, what indicates an age very likely older than two–three years^42^). The younger age categories were reclassified over time as follows: all juveniles PIT-tagged in one year were classified as young adults in the next two years; young adults with intermediate chin spot in one year were classified as old in the next year (those with dark spot were kept as young adults also in the next year).

### Genotyping

To explore relative genetic distances amongst individuals in the study population, we genotyped all PIT-tagged bats whose activity was recorded on monitored roosts. A tissue sample was taken from the wing membrane with a small biopsy-punch (2 mm^2^) and stored in 96% ethanol in freezer (− 20 °C) pending analysis. DNA was extracted with EchoLUTION Tissue DNA Micro Kit (Bioecho Life Sciences, GmbH). Using three multiplex PCR protocols, 12 microsatellite loci^43–46^ were screened (multiplex 1: H19, D15, E24, ES43-Mluc, D9; multiplex 2: C113, A24-Mluc, Paur6; multiplex 3: H29, G9, B15, B22). The PCR protocol required 4 μl of genomic DNA (~ 10–20 ng.μl^-1^), 0.25 μM of each primer (forward and reverse) and a 1 × PCRBIO HS Taq Mix Red Master mix (PCRBiosystems, Ltd.) in a 20 μl reaction volume. Fragments of multiplex 1 and 2 were amplified in the thermocycler TAdvanced (Biometra) at the following steps: initial denaturation at 95 °C for 5 min; 35 cycles of denaturation at 95 °C for 30 s; annealing at 58 °C for 30 s; extension at 72 °C for 90 s; and a final elongation step at 72 °C for 5 min. Multiplex 3 only differed in annealing temperature (55 °C). The fluorescent labelled PCR products (dyes ATTO 550, ATTO 565, 6-FAM, Yakima Yellow) were separated by capillary electrophoresis in an ABI 3730XL genetic analyser, and fragment lengths estimated relative to the size standard LIZ600 were determined using Geneious Prime 2019 software (Biomatters, Ltd.). Three loci exhibited some elevated frequency of null alleles (D15 and C113 10%; B22 30%) when tested using the Chakraborty et al.’s and Brookfield’s methods in the package ‘PopGenReport’ 3.0.4^47^ of the R 3.6.3 software^48^.

### Statistical analyses

To test the hypothesis that the frequency of nocturnal swarming activity of individuals of different sex and age categories come from the same distribution (i.e. temporal pattern of swarming during a night did not differ), we compared empirical cumulative distribution functions (ECDFs) for each pairwise combination of groups (six sex/age categories) by randomisation based two-sample tests. The most powerful test based on the DTS test statistic, which is the reweighted integral of the distance between the two ECDFs, with 1000 bootstrap iterations was performed in the R package ‘twosamples’ 1.1.1^49^. Furthermore, differences among individuals of different sex/age categories according to the mean and maximum number of their swarming events per night and roost were visualised using bean-plots^50^ in the R package ‘beanplot’ 1.2^51^ and determined by the non-parametric Kruskal–Wallis ANOVA with a post-hoc Dunn test with Bonferroni correction for multiple comparisons in the R package ‘FSA’ 0.8.30^52^.

An individual-based genetic distance measure was extracted from the PC eigenvalues of a principal coordinates analysis (PCoA) performed in the R package ‘adegenet’ 2.1.3^53^. This multivariate method considers the best approximation of a dissimilarity matrix of Euclidean and short distances, and its principal components optimise the representation of the squared pairwise distances between individuals. The relationship between individual genetic distance relative to other bats in the study population and their activity at roosts was examined by Spearman’s rank-order correlation using the R package ‘ggpubr’ 0.4.0^54^, which depicted a 95% confidence interval.

### Ethical statement

This study adhered to the Guidelines for the Treatment of Animals in Behavioral Research and Teaching^55^ and the legal requirements of the country (Slovakia) in which the work was performed. The protocol for this research was approved by the Ministry of the Environment of the Slovak Republic (4021/2017–6.3, 6169/2021–6.3) according to the decision of its own ethics review board. ARRIVE guidelines for reporting animal research have been followed as much as possible (https://arriveguidelines.org/).

## Results

Altogether, 59,622 activity events (an event was any presence of a bat at the cavity entrance, mainly emergence, swarming and entering the tree) of 281 individuals were recorded on 10 monitored roosts during a total of 261 nights across three summer seasons (Table 1). In average 241.7 (SE ± 18.8) events of an individual during 37.2 (± 2.5) nights were recorded within the whole study period (three seasons) or 118.1 (± 7.0) and 17.7 (± 0.7), respectively, during a season (Table 2). This activity was recorded on monitored roosts mostly in June, less at the turn of July–August (Fig. 2a).

**Table 1:** Summary statistics of the number of unique PIT-tagged individuals, nights with records and behavioural events associated with emergence/entering and swarming recorded at ten tree roosts of Daubenton’s bats during three seasons.

Roost	Individuals	Nights	Events
A	203	95	14,795
B	179	115	23,149
C	73	43	2528
D	21	9	69
E	12	20	491
F	149	85	7736
G	8	12	117
H	111	62	4140
I	99	54	4250
J	62	18	2347
Total	281	261	59,622

**Table 2:** Mean number of nights and events with standard error recorded per an individual and season (codes for sex and age categories: F = female, M = male, A = adult, O = old, Y = young, J = juvenile). Juveniles were not PIT-tagged in 2021.

Sex/age	Nights	Events	n_2019_	n_2020_	n_2021_
FA_O_	21.9 ± 1.1	133.2 ± 11.5	66	78	75
FA_Y_	21.3 ± 1.4	131.5 ± 12.3	54	33	46
FJ	9.1 ± 0.8	122.3 ± 24.6	27	30	–
MA_O_	5.2 ± 1.4	27.7 ± 8.0	2	7	6
MA_Y_	6.5 ± 1.5	39.5 ± 12.9	4	10	5
MJ	7.3 ± 0.7	68.0 ± 10.0	25	24	–

During individual nights, the first peak of swarming activity began on average 15 min after sunset and lasted for 30 min. This period included emerging events from the roost as well as repeated returns. Based on direct observations at the site, less frequent data prior to this period should represent unintentional recordings of individuals positioned in the roost entrance and waiting for dusk (bats typically aggregated at the entrance before emergence close to the sensitivity range of the PIT tag reader antenna) or some so-called ‘scouts’ that prematurely emerged and quickly returned due to high light intensity yet. Subsequently, activity rapidly decreased almost to zero before midnight, and then swarming gradually increased up to the second maximum, which occurred 75–30 min before sunrise when bats returned to roosts (Fig. 2b). However, separate analysis of sex/age categories found different frequency distribution patterns. First, a gradual increase of swarming activity from midnight toward sunrise was more pronounced in old than young adult females, the latter of which more closely resembled that of juveniles, which mostly swarmed during dawn. Secondly, swarming patterns strongly differed between old and young adult males categories which both swarmed at maternity roosts. Whereas young males’ activity was similar to female patterns, as they more likely roosted together, old males mostly swarmed around maternity roosts between crepuscular periods (Fig. 2c). Activity distributions in the categorised groups significantly differed in all pairwise combinations (*P* < 0.001).

**Figure 3:**
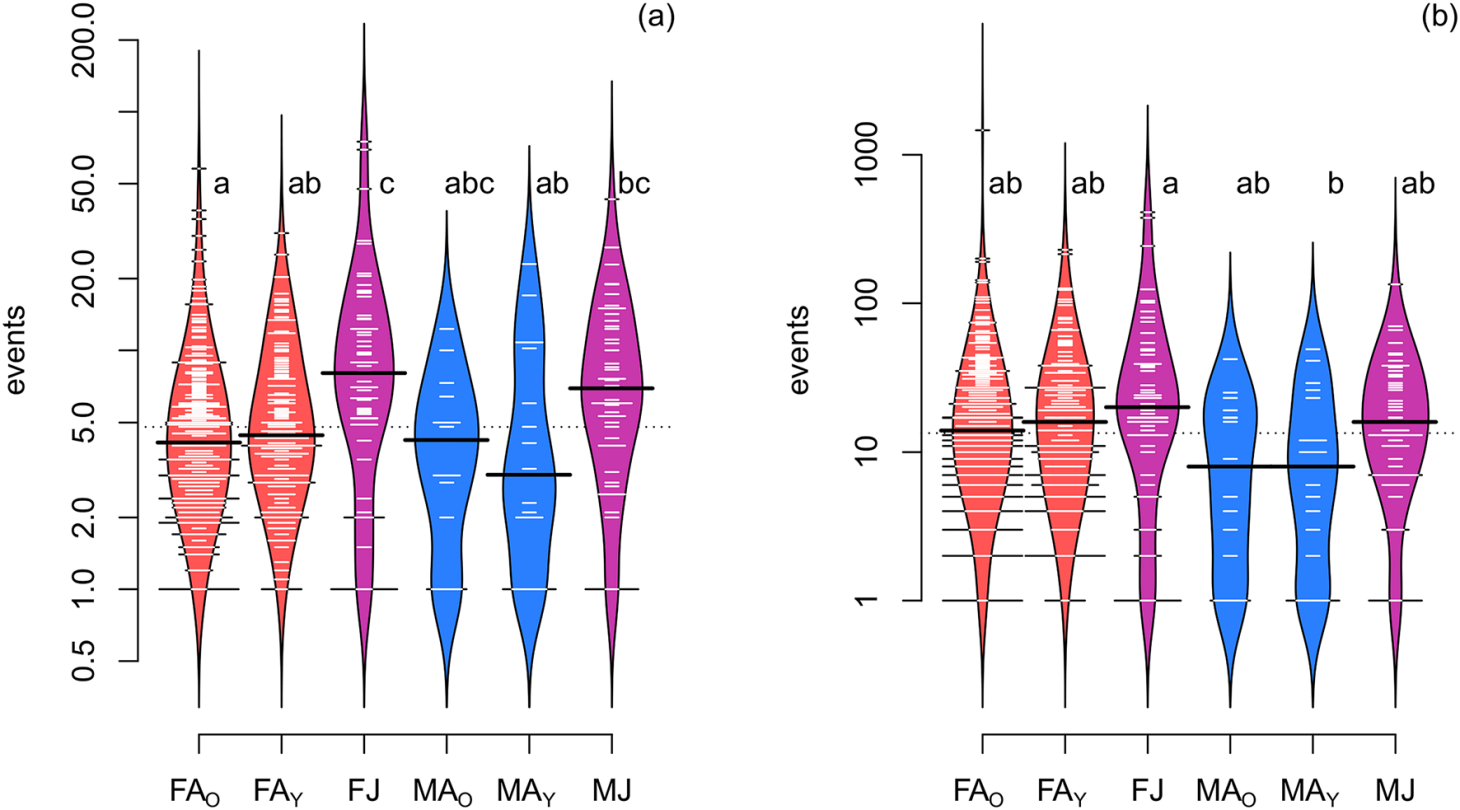
Relative swarming activity of individuals of different demographic categories of bats collected during three seasons calculated as (**a**) the mean number or (**b**) the maximum number of their events per night and roost (codes for sex and age categories: F = female, M = male, A = adult, O = old, Y = young, J = juvenile; categories are also colour coded). The distribution of individual observations in bean-plots (small horizontal lines) is shown by a kernel density shape and an median value (bold line). Different lower-case letters denote groups that significantly differ (*P* < 0.05) from the others according to post-hoc Dunn tests with Bonferroni correction.

**Figure 4:**
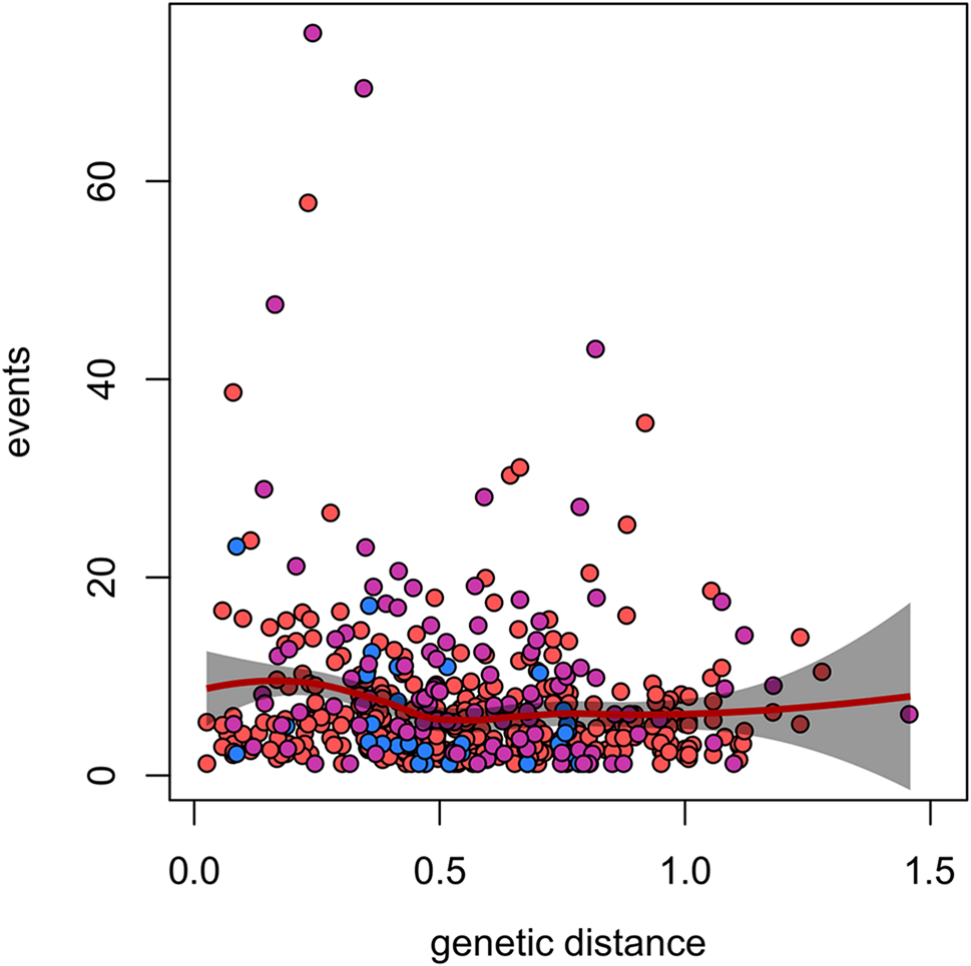
Spearman’s rank-order correlation between genetic distance based on PCoA components and relative swarming activity of individuals. Local polynomial regression curve fits the deterministic part of the variation in the data with 95% confidence interval. Different demographic categories of individuals (adult females, adult males, juveniles) are colour-coded as in Figs. 2 and 3.

Individual swarming activity within sex and age categories also significantly differed, either measured as the mean number (*χ*^2^ = 29.4, df = 5, *P* < 0.001) or the maximum number of events per night and roost (χ^2^ = 14.0, df = 5, *P* = 0.015). The number of events was higher in juveniles than adult females, and adult males showed the least activity (Fig. 3). Measures of genetic variability in the study population (missing data 1.4%) showed that the mean number of alleles per locus was 16.2 (range 4–25), and the expected heterozygosity was 0.84 (range 0.47–0.93). Genotypes of individuals demonstrated variations in the multivariate PCoA analysis (PC1 = 22%, PC2 = 18%), and sum of absolute eigenvalues of two first PC axes was used to estimate individual genetic distance. Relative activity of individuals negatively correlated with their mean genetic distance from the rest of the colony (*ρ* = − 0.11, *P* = 0.011; Fig. 4); however, no significant pattern was found when activity was analysed within demographic categories.

## Discussion

This study revealed significant variability in swarming patterns according to demographic characteristics of tree-dwelling Daubenton’s bats. Observed differences between individuals engaged in dawn swarming showed that this non-centralised decision-making about the position of their roost is even more complex^9^. The adaptive value of roost switching in bats is not strictly restricted to specific age or sex categories^56^; however, our novel results indicate the important role of these demographic characteristics in shaping cooperative swarming behaviour, which serves as a mechanism maintaining cohesion of bat groups during this process. In accordance with our hypothesis we found that adult females performed more swarming activity than adult males which accompanied maternity groups during nursing period. Similarly, we conformed that swarming activity patterns changed throughout individual’s lifespan and that kinship may play a role in the level of dawn swarming activity.

Swarming around a tree hollow in dim light conditions enhance the risk of predation^15,34^, thus light intensity is assumed to be the main factor forcing bats to return to the roosts^6^. Maintenance of this potentially dangerous and energetically demanding activity^31^ indicates that it should be driven by some individual benefits, presumably related to the matrilineal societies^17,36^. The main factors leading to formation of these groups are the high fidelity of adult females to the natal area, where they live with their progenitors for their entire lifetime^57^ and relative longevity of bats which allows them to form social bonds lasting multiple seasons^58^. Such formation opens the door for the evolution of sex-specific cooperative behaviour, such as dawn swarming, that provides social benefits for individual participants despite being energetically demanding and risky^31^. Therefore, it was not surprising that old females as a main part of the colony had a high number of swarming events as well as organised, increasing gradients of behavioural displays during night-time. Juveniles have to undergo various steps of the flight development to be able of full-featured flight^59^, thus we might expect that some stages are necessary also for swarming behaviour development. Because juveniles and younger females swarm mostly during dawn, we suggest this as a developmental stage which is necessary to learn and perform a defined pattern of behaviour in adulthood. Older experienced bats likely take a more active role in signalling about the position of roost. However, these differences between individuals may not purely follow age characteristics, but it may be consistent for a small set of individuals that play a central role in leading other bats to roosts^60^. The development of cooperative behaviour during the juvenile phase has an impact on social relationships in adulthood^61^. Bats must become independent from maternal care shortly after weaning^59^; thus, the elevated swarming activity of weaned juveniles is likely related to their learning necessity, together with benefits gained from colony membership. They should mainly learn the location of colony roosts^31,36^, and the time spent together with older bats may reflects cooperative social interactions^62^. In contrast, males exhibited the lowest amount of activity and did not participate in swarming shortly before sunrise, the time when decisions concerning roost position are mainly made. It confirms that old males are not a stable part of the colony^24^ and group cohesion is not a crucial issue for them. However, the female-like swarming pattern among younger males, which often remain in natal area during their first year, is in accordance with their higher affinity to the maternity colony explained by familiarity or mating options^26^.

The necessity for cooperation is especially emphasised in fission–fusion societies^10^, and among tree-dwelling bats, this is further enhanced by relatedness in social groups^63^. Albeit statistically weak, the negative correlation between genetic distance and swarming activity suggests the cooperative nature of this behaviour and indicates an important role of kinship in the formation of groups during the roost switching process. Similar to the kin-selection theory^64^, we can suggest that individuals more related to the rest of the colony likely give more attention to maintenance of group cohesion and thus invest more time in swarming activity^36^. The importance of relatedness in shaping social bonds depends on group size. Relatedness acts as a clue mostly in larger groups, whereas familiarity is sufficient for a smaller group^17^. The high number of PIT-tagged Daubenton’s bats (almost four hundred individuals) in our small study plot indicates large colony size what thus supports an effect of kinship in development of this cooperative behaviour. An open question remains whether observed patterns of swarming activity would be similar in smaller colonies of Daubenton’s bats or other tree-dwelling species.

Most swarming events in our data were in fact recorded at roosts that were preferred by females during their lactation. In this period, maintaining larger groups mirrored in an increased swarming activity is an energy-saving option to maintain stable roost temperature preventing torpor, which may slow down offspring development^11,65^. However, increasing swarming activity is also an excessive energy loss^31^ and a trade-off between costs and benefits for lactating females need to be considered. With the end of lactation, the mean group size decreases, and the colony disperses into more shelters^66^. The limited number of PIT-tag readers, which were only installed on the most frequently occupied roosts, could be a cause of the decline in the number of recorded swarming events in July. However, the second, smaller peak visible at the turn of July and August result to some extent from intensive swarming activity of weaned juveniles around the well-established long-term roosts of maternity colony in the study area^35^. They might potentially learn the position of roost in a colony home range^31^, also with guidance from their mothers^67^. Despite current technological limitations, which did not allow us to record the activity of individuals at a larger distance from the roost (around tree canopies) or at all potential roosts that can be visited and occupied in the colony’s home-range^9^, our study provides the first in-depth insight into this impressive but little explored behaviour of bats. This complex cooperative behaviour in non-centralised decision-making process during the roost site selection highlights also the importance of sensitive forest harvesting interventions, respecting roosting habits of tree-dwelling bats.

## Supplementary Information

Supplementary Information 1.

Supplementary Information 2.

## Data Availability

Datasets used in this study are included in Supplementary Information files (recorded swarming events “events.csv” and genotypes “genotypes.csv” of PIT-tagged bats).

## References

[1] Green PA, Brandley NC, Nowicki S (2020). Categorical perception in animal communication and decision-making. Behav. Ecol..

[2] Petak I, Vonk J, Shackelford T (2019). Ritualization. Encyclopedia of Animal Cognition and Behavior.

[3] Fernandez AA, Fasel N, Knörnschild M, Richner H (2014). When bats are boxing: Aggressive behaviour and communication in male Seba’s short-tailed fruit bat. Anim. Behav..

[4] van Schaik J, Janssen R, Bosch T, Haarsma AJ, Dekker JJ, Kranstauber B (2015). Bats swarm where they hibernate: Compositional similarity between autumn swarming and winter hibernation assemblages at five underground sites. PLoS ONE.

[5] Vaughan T, O’Shea T (1976). Roosting ecology of the Pallid bat, *Antrozous pallidus*. J. Mammal..

[6] Kunz TH, Kunz TH (1982). Roosting ecology. Ecology of Bats.

[7] Kaňuch P (2007). Evening and morning activity schedules of the noctule bat (*Nyctalus noctula*) in Western Carpathians. Mammalia.

[8] Naďo L, Kaňuch P (2015). Swarming behaviour associated with group cohesion in tree-dwelling bats. Behav. Processes..

[9] Zelenka Z, Kasanický T, Budinská I, Kaňuch P (2020). An agent-based algorithm resembles behaviour of tree-dwelling bats under fission–fusion dynamics. Sci. Rep..

[10] Aureli F (2008). Fission-fusion dynamics: New research frameworks. Curr. Anthropol..

[11] Willis CKR, Brigham RM (2004). Roost switching, roost sharing and social cohesion: Forest-dwelling big brown bats, *Eptesicus fuscus*, conform to the fission-fusion model. Anim. Behav..

[12] Dietz C, Kiefer A (2016). Bats of Britain and Europe.

[13] Kerth G, Weissmann G, König B (2001). Day roost selection in female Bechstein’s bats. Oecologia.

[14] Reckardth K, Kerth G (2007). Roost selection and roost switching of female Bechstein’s bats. Oecologia.

[15] Mikula P, Hromada M, Tryjanowski P (2013). Bats and swifts as food of the European kestrel (*Falco tinnunculus*) in small town in Slovakia. Ornis Fennica.

[16] Popa-Lisseanu AG, Bontadina F, Mora O, Ibáñez C (2008). Highly structured fission-fusion societies in an aerial-hawking carnivorous bat. Anim. Behav..

[17] Patriquin KJ, Palstra F, Leonard ML, Broders HG (2013). Female northern myotis (*Myotis septentrionalis*) that roost together are related. Behav. Ecol..

[18] Sherman PW (1977). Nepotism and the evolution of alarm calls. Science.

[19] Kerth G, Almasi B, Ribi N, Thiel D, Lüpold S (2003). Social interactions among wild female Bechstein’s bats (*Myotis bechsteinii*) living in a maternity colony. Acta Ethol..

[20] Dietz M, Kalko EKV (2007). Reproduction affects flight activity in female and male Daubenton’s bats, *Myotis daubentonii*. Can. J. Zool..

[21] Nelson RJ, Kriegsfeld LJ (2017). An Introduction to Behavioral Endocrinology.

[22] Choleris E, Kavaliers M (1999). Social learning in animals: Sex differences and neurobiological analysis. Pharmacol. Biochem. Behav..

[23] McCracken GF, Wilkinson GS, Crichton EG, Krutzsch PH (2000). Bat mating systems. Reproductive Biology of Bats.

[24] Safi K (2008). Social bats: The males’ perspective. J. Mammal..

[25] Linton DM, Macdonald DW (2019). Roost composition and sexual segregation in a lowland population of Daubenton’s bats (*Myotis daubentonii*). Acta Chiropterol..

[26] Ružinská R, Kaňuch P (2021). Adult males in maternity colonies of Daubenton’s bat, *Myotis daubentonii*: What are they?. Mammalia.

[27] Barale CL, Rubenstein DI, Beehner JC (2015). Juvenile social relationships reflect adult patterns of behavior in wild geladas. Am. J. Primatol..

[28] McFarland D (2006). A Dictionary of Animal Behaviour.

[29] Ratcliffe J, Hofstede H (2005). Roosts as information centres: Social learning of food preferences in bats. Biol. Lett..

[30] Fernandez AA, Burchardt LS, Nagy M, Knörnschild M (2021). Babbling in a vocal learning bat resembles human infant babbling. Science.

[31] Wilkinson GS (1992). Information transfer at evening bat colonies. Anim. Behav..

[32] Vesterinen EJ (2016). What you need is what you eat? Prey selection by the bat *Myotis daubentonii*. Mol. Ecol..

[33] Todd VLG, Waters DA (2017). Small scale habitat preferences of *Myotis daubentonii*, *Pipistrellus pipistrellus*, and potential aerial prey in an upland river valley. Acta Chiropterol..

[34] Kaňuch P (2005). Roosting and population ecology of three syntopic tree-dwelling bat species (*Myotis nattereri, M. daubentonii* and *Nyctalus noctula*). Biologia.

[35] Lučan RK, Hanák V (2011). Population ecology of *Myotis daubentonii* (Mammalia: Chiroptera) in South Bohemia: Summary of two long-term studies: 1968–1984 and 1999–2009. Acta Soc. Zool. Bohem..

[36] Patriquin KJ, Ratcliffe JM, Ortega J (2016). Should I stay or should I go? Fission-fusion dynamics in bats. Sociality in Bats.

[37] Bogdanowicz W (1994). *Myotis daubentonii*. Mamm. Species.

[38] Rigby EL, Aegerter J, Brash M, Altringham JD (2012). Impact of PIT tagging on recapture rates, body condition and reproductive success of wild Daubenton’s bats (*Myotis daubentonii*). Vet. Rec..

[39] Henry M, Thomas DW, Vaudry R, Carrier M (2002). Foraging distances and home range of pregnant and lactating Little brown bats (*Myotis lucifugus*). J. Mammal..

[40] Brunet-Rossinni AK, Wilkinson GS, Kunz TH, Parsons S (2009). Methods for age estimation and the study of senescence in bats. Ecological and Behavioral Methods for the Study of Bats.

[41] Richardson PW (1994). A new method of distinguishing Daubenton’s bats (*Myotis daubentonii*) up to one year old from adults. J. Zool..

[42] Haarsma A, van Alphen J (2009). Chin-spot as an indicator of age in pond bats. Lutra.

[43] Burland TM, Barratt EM, Racey PA (1998). Isolation and characterization of microsatellite loci in the brown long-eared bat, *Plecotus auritus*, and cross-species amplification within the family Vespertilionidae. Mol. Ecol..

[44] Castella V, Ruedi M (2000). Characterization of highly variable microsatellite loci in the bat *Myotis myotis* (Chiroptera: Vespertilionidae). Mol. Ecol..

[45] Kerth G, Safi K, König B (2002). Mean colony relatedness is a poor predictor of colony structure and female philopatry in the communally breeding Bechstein’s bat (*Myotis bechsteinii*). Behav. Ecol. Sociobiol..

[46] Jan C, Dawson DA, Altringham JD, Burke T, Butlin RK (2012). Development of conserved microsatellite markers of high cross-species utility in bat species (Vespertilionidae, Chiroptera, Mammalia). Mol. Ecol. Resour..

[47] Gruber, B. & Adamack, A. PopGenReport: A simple framework to analyse population and landscape genetic data. R package version 3.04. https://cran.r-project.org/package=popgenreport (2019).

[48] R Core Team. R: A language and environment for statistical computing (R Foundation for Statistical Computing, 2020).

[49] Dowd, C. twosamples: Fast permutation based two sample tests. R package version 1.1.1. https://cran.r-project.org/package=twosamples (2020).

[50] Kampstra P (2008). Beanplot: A boxplot alternative for visual comparison of distributions. J. Stat. Soft. Code Snippets.

[51] Kampstra, P. beanplot: Visualization via beanplots (like boxplot/stripchart/violin plot). R package version 1.2. https://cran.r-project.org/package=beanplot (2014).

[52] Ogle, D. H., Wheeler, P. & Dinno, A. FSA: Fisheries stock analysis. R package version 0.8.30. https://github.com/droglenc/FSA (2020).

[53] Jombart T (2008). adegenet: A R package for the multivariate analysis of genetic markers. Bioinformatics.

[54] Kassambara, A. (2020) ggpubr: ‘ggplot2’ based publication ready plots. R package version 0.4.0. https://cran.r-project.org/package=ggpubr (2020).

[55] Animal Behaviour. Guidelines for the treatment of animals in behavioural research and teaching. *Anim. Behav.* 159, I–X (2020). 10.1006/anbe.1999.1349 10640387

[56] Russo DL, Cistrone L, Jones G, Mazzoleni S (2004). Roost selection by barbastelle bats (*Barbastella barbastellus*, Chiroptera: Vespertilionidae) in beech woodlands of central Italy: Consequences for conservation. Biol. Conserv..

[57] Arnold BD, Wilkinson GS (2015). Female natal philopatry and gene flow between divergent clades of pallid bats (*Antrozous pallidus*). J. Mammal..

[58] Barclay RMR, Harder LD, Kunz TH, Fenton MB (2003). Life histories of bats: Life in the slow lane. Bat Ecology.

[59] Sun D (2020). Behavioural patterns and postnatal development in pups of the Asian parti-coloured bat, *Vespertilio sinensis*. Animals.

[60] Mavrodiev P, Fleischmann D, Kerth G, Schweitzer F (2021). Quantifying individual influence in leading-following behavior of Bechstein’s bats. Sci. Rep..

[61] Bekoff M (1972). The development of social interaction, play, and metacommunication in mammals: An ethological perspective. Q. Rev. Biol..

[62] Dunbar RIM, Shultz S (2010). Bondedness and sociality. Behaviour.

[63] Kerth G, Perony N, Schweitzer F (2011). Bats are able to maintain long-term social relationships despite the high fission-fusion dynamics of their groups. Proc. R. Soc. B.

[64] Hamilton WD (1964). The genetical evolution of social behaviour. I. J. Theor. Biol..

[65] Ruczyński I, Bartoń KA (2020). Seasonal changes and the influence of tree species and ambient temperature on the fission-fusion dynamics of tree-roosting bats. Behav. Ecol. Sociobiol..

[66] Červený J, Bürger P, Hanák V, Horáček I, Gaisler J (1989). Density and structure of the bat community occupying an old park at Žihobce (Czechoslovakia). European Bat Research 1987.

[67] Ripperger S (2019). Proximity sensors on common noctule bats reveal evidence that mothers guide juveniles to roosts but not food. Biol. Lett..

